# *Sg*trCypB Formulated with Freund’s Adjuvant Confers Protective Immunity Against *S. globosa* and Partial Cross-Protection Against *S. schenckii* Infections in Mice

**DOI:** 10.3390/jof12090706

**Published:** 2026-09-21

**Authors:** Ling Hu, Baicheng Deng, Yuyan Hong, Meizhen Zhong, Xiaoyu Zhi, Xuchu Hu, Huaiqiu Huang

**Affiliations:** 1 Department of Dermatology and Venereology, The Third Affiliated Hospital of Sun Yat-Sen University, Guangzhou 510600, China; huling8@mail.sysu.edu.cn (L.H.); hongyy6@mail2.sysu.edu.cn (Y.H.); zhongmzh8@mail2.sysu.edu.cn (M.Z.); zhixy5@mail2.sysu.edu.cn (X.Z.); 2 Department of Parasitology, Zhongshan School of Medicine, Sun Yat-Sen University, Guangzhou 510000, China; dengbch@mail2.sysu.edu.cn

**Keywords:** sporotrichosis, *Sporothrix globosa*, Cyclophilin B, recombinant protein vaccine, protective immunity, cross-protection, Adjuvant

## Abstract

*Sporothrix globosa* is the predominant cause of sporotrichosis in China, highlighting the need for effective vaccine strategies. Cyclophilins from pathogenic microorganisms have emerged as potential vaccine antigens. We previously generated a high-yield, high-purity recombinant truncated *S. globosa* cyclophilin B protein (*Sg*trCypB) retaining its functional domain. Here, BALB/c mice were immunized subcutaneously with *Sg*trCypB alone or formulated with Freund’s adjuvant to evaluate immunogenicity. Only the adjuvant-formulated vaccine induced a high antibody titer (1:51,200). Then, the infection-challenge experiments compared the *Sg*trCypB formulated with Freund’s adjuvant and PBS formulated with Freund’s adjuvant groups. Following challenge, *Sg*trCypB plus adjuvant reduced cutaneous lesion size (0.6 vs. 1.0 cm at week 1) without significantly affecting ulcer incidence. In systemic *S. globosa* infection, vaccination increased survival to 90% versus 40% in controls and reduced organ fungal burdens. Partial cross-protection was observed against *S. schenckii*, with 60% versus 20% survival and lower fungal burdens in the kidneys and lungs. Protection against *C. albicans* was minimal. These findings identify *Sg*trCypB as a promising protective antigen against *S. globosa* and *S. schenckii* and support further evaluation with clinically applicable adjuvants.

## 1. Introduction

Sporotricosis is a subcutaneous fungal infection mainly caused by the pathogenic clade of the genus *Sporothrix* [1]. Its prevalence in China has steadily increased over the past decade, with *Sporothrix globosa* (*S. globosa*) accounting for the majority of cases, which is primarily transmitted through sapronotic routes, meaning infection typically occurs via traumatic inoculation of contaminated plant debris or soil during outdoor activities. The disease is widely distributed across the country, with the northeastern provinces (Jilin, Liaoning, and Heilongjiang) being the most endemic regions. A notably higher incidence has been observed during colder seasons in these areas [2,3,4]. Clinical manifestations include fixed cutaneous, lymphocutaneous, disseminated cutaneous, and extracutaneous forms [5,6,7,8]. Conventional treatment for sporotrichosis requires prolonged antifungal therapy, which is often complicated by adverse drug reactions, the emergence of antifungal resistance [9], and frequent recurrence, particularly in immunocompromised patients [10,11]. These challenges have prompted the exploration of alternative strategies for the prevention and management of sporotrichosis, including the development of vaccines [12,13]. Unlike conventional antifungal therapy, vaccination has the potential to combating sporotrichosis, with the potential to prevent disease before systemic dissemination occurs. Vaccination has the potential to prevent infection or limit disease progression [14]. Accordingly, increasing attention has been directed toward developing subunit vaccines based on defined microbial components because of their favorable safety profiles [15,16,17,18,19]. Recombinant subunit vaccines, as safe, specific, and scalable immunotherapeutic strategies, are expected to overcome the limitations of conventional antifungal treatments [20].

The protective efficacy of various cell wall proteins (CWPs) from *Sporothrix* spp. has been evaluated in murine models. Among these, Gp70 is an immunodominant cell wall antigen of *Sporothrix*. Anti-Gp70 antibodies, as well as selected Gp70-derived epitopes and peptides, have demonstrated protective activity and reduced infection-associated mortality [21,22]. Other candidate antigens, including enolase, Gp60, Hsp90, Hap1, Pap1, Pap2 and Hsp60 have also shown varying degrees of protective activity in vitro and/or in vivo [23,24,25,26]. To date, studies evaluating vaccine-mediated protection against *S. globosa* remain limited, which evaluated the protective efficacy of a recombinant phage displaying Gp70 epitopes, and reported a 60% reduction in mortality together with enhanced Th1 and Th17 responses. Inactivated *S. globosa* provided 50% protection in the same study [27]. Furthermore, Gp60 immunization has been evaluated against cutaneous *S. schenckii* infection, which accelerated lesion healing and reduced the incidence of ulceration in BALB/c mice, although it did not significantly alter the degree of inflammation [23]. However, protective immunity against cutaneous *S. globosa* infection remains poorly characterized.

Cyclophilins have emerged as potential protective antigens against several infectious diseases. Previous studies have demonstrated protective effects of cyclophilin-based immunization against infections such as schistosomiasis, toxoplasmosis, leishmaniasis, and sepsis [28,29,30,31]. In *Cryptococcus neoformans*, the cyclophilin A homologs Cpa1 and Cpa2 play overlapping roles in fungal growth, mating, virulence, and infection. The Cpa1-Cpa2 double mutant exhibits severely attenuated virulence and impaired growth at elevated temperatures [32], suggesting an important role for cyclophilins in fungal pathogenicity. Previously, we demonstrated that cyclophilin B is localized on the surface of *S. globosa* [33], where it is potentially accessible to host immune recognition. However, whether S. globosa cyclophilin B can induce protective immunity has not been established. We subsequently expressed a recombinant truncated form of *S. globosa* cyclophilin B protein (*Sg*trCypB) at high yield [34]. This recombinant protein retains the characteristic peptidyl-prolyl cis-trans isomerase (PPIase) activity of its functional domain. Together with previous linear B-cell epitope prediction indicating that most predicted epitopes are located within the extracellular PPIase functional domain [33], these findings provide a rationale for evaluating *Sg*trCypB as a candidate protein vaccine antigen.

In the present study, we immunized the recombinant *Sg*trCypB protein to immunize BALB/c mice to evaluate its protective efficacy of *Sg*trCypB protein against both cutaneous and systemic *S. globosa* infection. The *Sg*CypB protein s shares approximately 99% sequence identity with CypB from *Sporothrix schenckii*, whereas no homologous gene has been identified in *Candida albicans* [33]. Therefore, *S. schenckii* and *C. albicans* were selected as heterologous challenge organisms to evaluate whether CypB could serve as a common protective antigen against *Sporothrix* infection.

## 2. Materials and Methods

### 2.1. Fungal Strains, Culture Conditions, and Preparation of Fungal Suspensions

*Sporothrix globosa* SS01, *Sporothrix schenckii* CBS 359.36, and *Candida albicans* ZSSY2021001 (all strains were obtained from the Department of Dermatology, the Third Affiliated Hospital of Sun Yat-Sen University) were used in this study. *Sporothrix* globosa and *Sporothrix schenckii* were inoculated onto Sabouraud dextrose agar (SDA; Solarbio, Beijing, China) slants and incubated at 25 °C for 7 days, then transferred to potato dextrose agar (PDA; Solarbio, Beijing, China) and cultured for an additional week to promote conidial formation. *C. albicans* was cultured on SDA or PDA at 37 °C for 24–48 h. Fungal colonies were gently resuspended in 2 mL of sterile phosphate-buffered saline (PBS; Solarbio, Beijing, China), then filtered through a 40 μm cell strainer to remove hyphal fragments. The filtrate was centrifuged at 12,000× *g* for 5 min, and the pellet was washed three times with sterile PBS. The final pellet was resuspended in 1 mL of PBS, and the cell concentration was determined using a hemocytometer. The suspension was adjusted to the required concentrations for subsequent infection experiments, and viability was confirmed by colony-forming unit (CFU) counts on agar plates. Only suspensions with viability >87% were used for challenge experiments, as described in established *Sporothrix* murine models [35].

### 2.2. Production of Recombinant SgtrCypB

The recombinant truncated *S. globosa* cyclophilin B protein (*Sg*trCypB) was produced as previously described [34]. Briefly, the gene fragment encoding the extracellular PPIase domain of *Sg*CypB was cloned into the pET-30a(+) vector and expressed in *E. coli* BL21(DE3). Protein expression was induced with 0.5 mM IPTG at 25 °C for 6 h. The His-tagged recombinant protein was purified by Ni-NTA affinity chromatography followed by gel filtration. Protein purity was assessed by SDS-PAGE and identity was confirmed by Western blot using anti-His antibody.

### 2.3. Immunization Protocol

Specific pathogen-free (SPF) male BALB/c mice (6–8 weeks old) were randomly divided (*n* = 10 per group for one type of infection). For the antigen-only groups, mice received three subcutaneous injections of recombinant *Sg*trCypB protein (100 μg per injection, without adjuvant) at weekly intervals. Blood samples were collected from the inner canthus before immunization and one week after each injection to monitor antibody responses. For the adjuvant-formulated groups, the immunization group (*Sg*trCypB + Freund’s adjuvant, SC + FA) and the control group (PBS + Freund’s adjuvant, PBS + FA). Mice in the SC + FA group were immunized subcutaneously with 100 μg of recombinant *Sg*trCypB protein, emulsified in an equal volume of Complete Freund’s Adjuvant (CFA; Sigma-Aldrich, St. Louis, MO, USA) for the primary immunization. Two booster immunizations were administered at two-week intervals with 50 μg of the protein emulsified in an equal volume of Incomplete Freund’s Adjuvant (IFA; Sigma-Aldrich, St. Louis, MO, USA). Control mice received an equal volume of PBS mixed with the corresponding Freund’s adjuvant on the same schedule. The two booster immunizations at two-week intervals is consistent with standard Freund‘s adjuvant protocols [36,37,38,39,40]. Blood samples were collected from the inner canthus before the initial immunization and two weeks after each immunization. Sera were separated from blood by centrifugation and stored at –80 °C until analysis.

### 2.4. Enzyme-Linked Immunosorbent Assay (ELISA)

Antibody titers were determined by enzyme-linked immunosorbent assay (ELISA). Briefly, 96-well microtiter plates (Nunc MaxiSorp; Thermo Fisher Scientific, Waltham, MA, USA) were coated with 100 µL/well of recombinant SgtrCypB protein (5 µg/mL in carbonate-bicarbonate buffer, pH 9.6) and incubated overnight at 4 °C. After five washes with PBST (PBS containing 0.05% Tween-20), the plates were blocked with 200 µL/well of PBST containing 5% skim milk and 1% BSA for 2 h at 37 °C, followed by another five washes. Mouse sera were serially two-fold diluted in PBST supplemented with 0.5% skim milk and 0.1% BSA, starting from an initial dilution empirically determined to bracket the endpoint titer; 100 µL of each dilution was added to duplicate wells and incubated for 2 h at 37 °C. Blank wells received PBST alone. After washing, horseradish peroxidase-conjugated goat anti-mouse IgG (1:20,000 dilution in PBST with 0.5% skim milk and 0.1% BSA) was added (100 µL/well) and incubated for 1 h at 37 °C. Following a final wash step, 200 µL/well of TMB substrate was added and incubated for 15 min at 37 °C in the dark. The reaction was stopped with 50 µL/well of 2 M H_2_SO_4_, and absorbance was read at 450 nm. The antibody titer was defined as the reciprocal of the highest serum dilution that yielded an OD_450_ value at least twice that of the blank control. Mice were challenged with the fungal inoculum when serum antibody titers in the immunized group reached 1:51,200 [41,42,43].

### 2.5. Fungal Challenge and Infection Models

Male, specific-pathogen-free (SPF) BALB/c mice (6–8 weeks old, weighing 20–25 g) were obtained from the Laboratory Animal Center of Sun Yat-sen University. Mice were housed under controlled conditions (22 ± 2 °C, 12 h light/dark cycle) with free access to food and water. All animals were acclimated for 7 days before the start of experiments.

For the cutaneous infection model, mice were anesthetized using inhaled isoflurane (3% for induction, 1.5–2% for maintenance, in 100% oxygen) delivered via a rodent anesthesia machine. Anesthesia depth was confirmed by the absence of a pedal withdrawal reflex before any procedure. The dorsal region was shaved. A 0.1 mL suspension of *S. globosa* containing 1 × 10^8^ CFU/mL was injected subcutaneously into the back. Lesion development was monitored, and the diameter of skin lesions was measured weekly using a digital caliper for four weeks. Ulcer formation was recorded. At the end of the observation period, skin lesion tissues were collected for histopathological analysis.

For systemic infection models, mice were challenged intravenously via the tail vein with 0.1 mL of fungal inocula as follows: *S. globosa* (1 × 10^7^ CFU/mL), *S. schenckii* (5 × 10^6^ CFU/mL), or *C. albicans* (5 × 10^5^ CFU/mL). Mice were monitored daily for morbidity and mortality over a four-week period. Survival curves were recorded.

All animal experiments were approved by the laboratory animal care and ethic committee of Guangdong Laidi Biomedical Research Institute Co., Ltd. (approval number: 2024007-1, 10 January 2024). All procedures were performed in accordance with the guidelines for the care and use of laboratory animals.

### 2.6. Histopathological Examination and Periodic Acid–Schiff Staining

Skin lesions and organ tissues (liver, kidney and lung) were fixed in 4% paraformaldehyde for 48 h. Tissues were then dehydrated through a graded ethanol series, embedded in paraffin, and sectioned at 4–5 μm thickness. For hematoxylin and eosin (H&E) staining, sections were deparaffinized in xylene, rehydrated through a graded ethanol series to distilled water, stained with hematoxylin for 5 min, differentiated in acid–alcohol, blued in tap water, and counterstained with eosin for 10 min. After dehydration and clearing, sections were mounted with neutral resin.

For periodic acid–Schiff (PAS) staining, deparaffinized and rehydrated sections were oxidized in 1% periodic acid for 10 min at room temperature, rinsed in distilled water, and then stained with Schiff‘s reagent for 10 min at room temperature in the dark. Sections were subsequently rinsed in three changes of sodium bisulfite solution (2 min each), washed in running tap water for 5 min, counterstained with hematoxylin for 1 min, dehydrated through graded ethanol, cleared in xylene, and mounted with neutral resin.

All stained sections were observed under a light microscope, and images were captured for analysis. Histopathological changes, including inflammatory infiltration and the presence of fungal elements, were evaluated by a pathologist blinded to the experimental groups.

### 2.7. Statistical Analysis

All statistical analyses were performed using GraphPad Prism 8.4. For longitudinal antibody titer data measured from the same animals at multiple time points, a repeated-measures two-way ANOVA was performed. Post-hoc comparisons were conducted using Šídák’s multiple comparisons test. Survival curves were analyzed using the log-rank (Mantel–Cox) test. For other quantitative data, normality was assessed using the Shapiro–Wilk test. Comparisons between two groups were conducted using unpaired Student’s t-test for parametric data or the Mann–Whitney U test for non-parametric data, as appropriate. Normally distributed data are presented as the mean ± SEM, whereas non-normally distributed data are presented as the median with interquartile range (IQR). A two-sided *p*-value < 0.05 was considered statistically significant. Data are presented as mean ± standard error of the mean (SEM). A *p*-value < 0.05 was considered statistically significant.

## 3. Results

### 3.1. Freund’s Adjuvant Formulation Enhances SgtrCypB-Specific Antibody Responses

**Figure 1:**
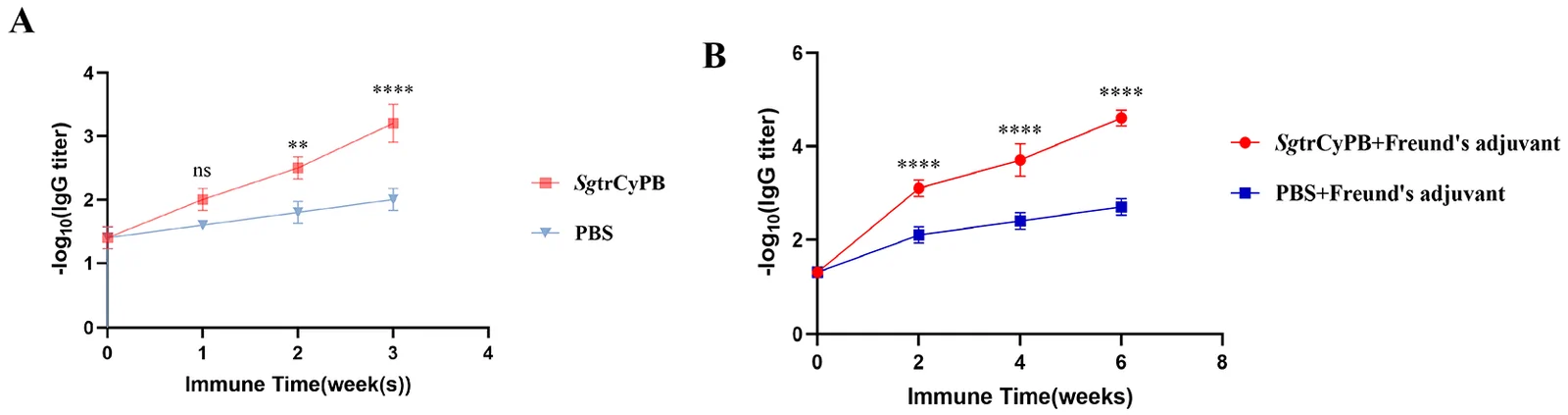
Kinetics of *Sg*trCypB-specific serum antibody responses in immunized mice. BALB/c mice were immunized with *Sg*trCypB alone at weekly intervals (**A**) or with Freund’s adjuvant at biweekly intervals (**B**). Serum antibody titers were measured by ELISA at the indicated time points. After three weekly injections of SgtrCypB (100 μg each), titers reached only 1:3200. In contrast, following three biweekly immunizations with the adjuvant-formulated protein (100 μg for the primary, 50 μg for the boosts), titers increased progressively to 1:51,200. Data are mean ± SEM (*n* = 5). Statistical analysis was performed using two-way repeated-measures ANOVA with Šídák’s post-hoc test (**, *p* < 0.01, ****, *p* < 0.0001; ns, not significant).

To evaluate the intrinsic immunogenicity of *Sg*trCypB, we first immunized BALB/c mice with the recombinant *Sg*trCypB alone. Using a weekly immunization regimen (three injections of 100 μg each), we observed that *Sg*trCypB alone induced only a weak antibody response. Two-way repeated-measures ANOVA revealed a significant interaction between treatment and time (*p* < 0.01). At week 1, serum antibody levels did not differ significantly from the PBS control group. Titers gradually increased to 1:800 by week 2 and reached a peak of 1:3200 at week 3, with significant differences observed at week 2 and 3 (*p* < 0.01 and *p* < 0.0001, respectively) (Figure 1A). Given the limited immunogenicity of the protein alone, we proceeded to evaluate whether formulation with an adjuvant could enhance the antibody response. When *Sg*trCypB was formulated with Freund‘s adjuvant and administered at biweekly intervals (100 μg for the primary dose, followed by two 50 μg boosts), two-way repeated-measures ANOVA again revealed a significant interaction between treatment and time (*p* < 0.0001). Antibody titers increased markedly after each injection, reaching 1:1600 at week 2, 1:12,800 at week 4, and 1:51,200 at week 6, with significant differences from the control group observed from week 2 onward (*p* < 0.0001 for all time points) (Figure 1B). These results indicated that the recombinant *Sg*trCypB alone was poorly immunogenic, and that adjuvant formulation was essential for eliciting high-titer antibody responses.

Given that both *Sg*trCypB and *Mycobacterium tuberculosis* (*M. tb*) Peptidyl-prolyl isomerase B (PpiB) belong to the cyclophilin family [44], and that Freund’s adjuvant contains mycobacterial components, we performed a sequence alignment between *Sg*trCypB and *M. tb* PpiB (Rv2582) to assess the potential for cross-reactivity. As shown in Supplementary Figure S1, the two proteins shared less than 35% sequence identity at the amino acid level, making significant antibody cross-reactivity unlikely.

### 3.2. SgtrCypB Formulated with FA Immunization Attenuates Cutaneous S. globosa Infection

**Figure 2:**
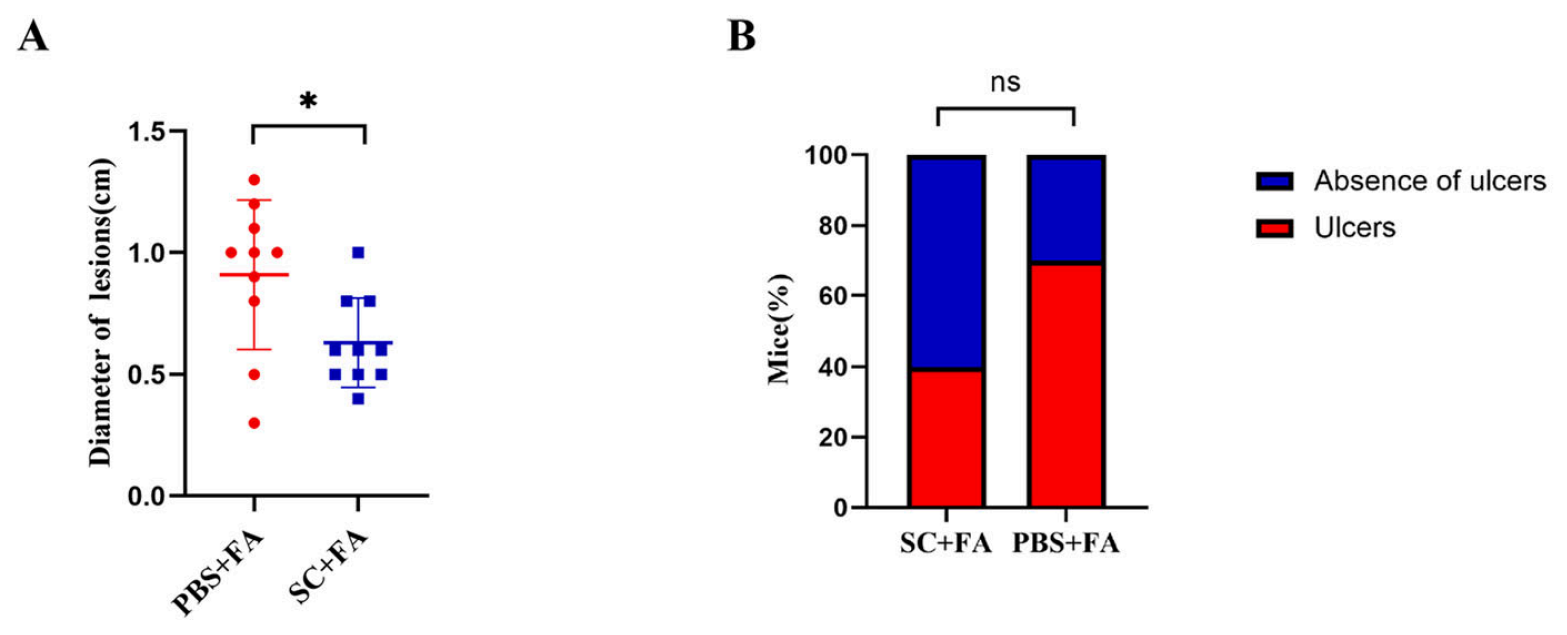
Recombinant *Sg*trCypB protein formulated with FA immunization induced protective immunity against cutaneous *S. globosa* infection in mice. BALB/c mice were immunized with recombinant *Sg*trCypB protein emulsified in Freund’s adjuvant (SC + FA group) or PBS with adjuvant (PBS + FA control group). (**A**) One week post-subcutaneous challenge with *S. globosa* (1 × 10^7^ CFU), the median diameter of skin lesions was significantly smaller in the SC + FA group (0.6 cm) than in the PBS + FA group (1.0 cm). Data are median ± IQR (*n* = 10 per group), Mann–Whitney U test (*p* = 0.0310, *n* = 10 per group). (**B**) Over the four-week observation period, the incidence of ulcer formation was 40% (4/10) in the SC + FA group and 70% (7/10) in the PBS + FA group; the difference was not statistically significant (*p* = 0.3700). Data are percentage (*n* = 10 per group), Fisher‘s exact test (*, *p* < 0.05; ns, not significant).

**Figure 3:**
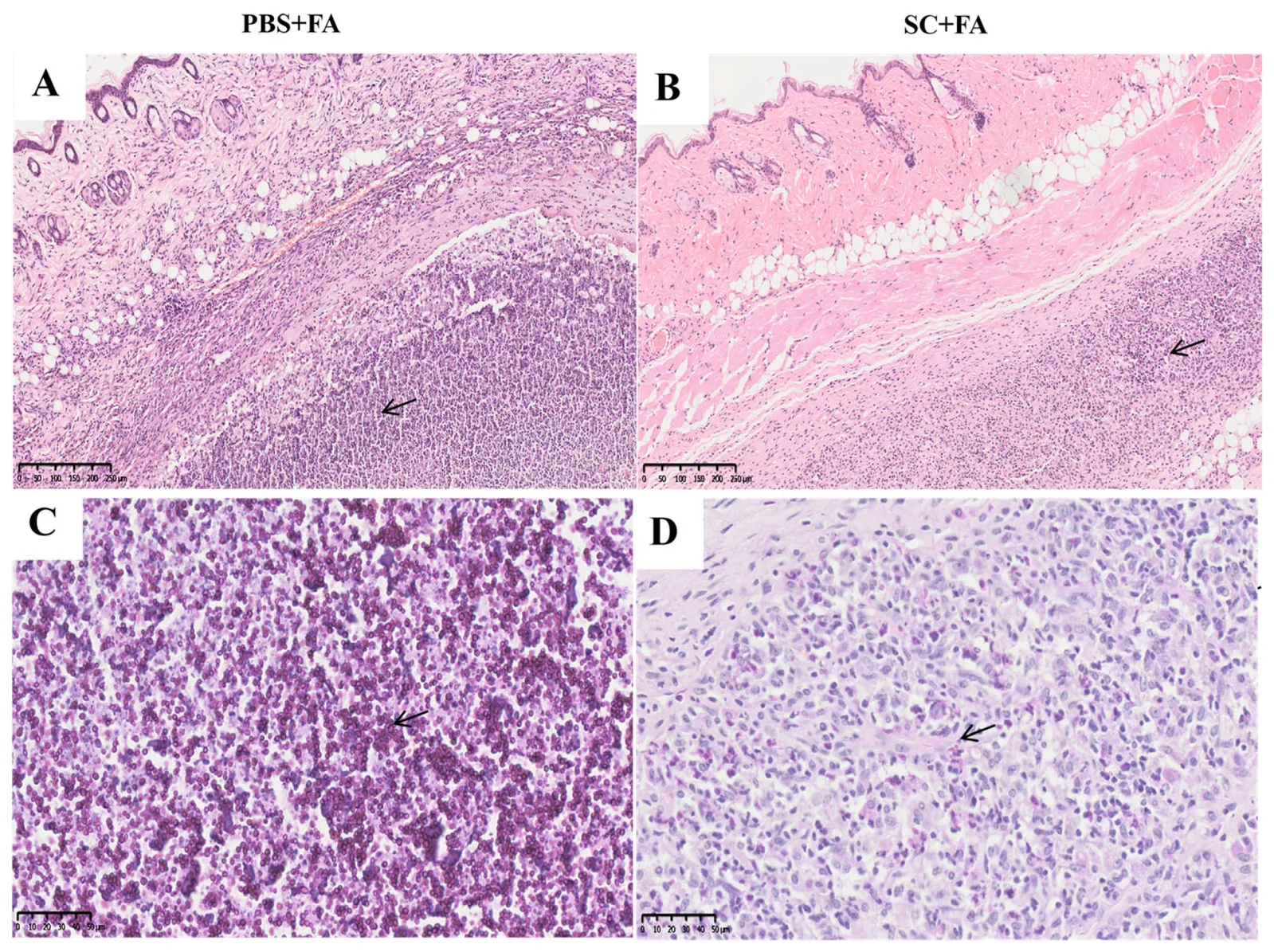
Histopathological examination of skin lesion. Skin tissues were collected at week 4 post-infection and stained with (**A**,**B**) hematoxylin and eosin (H&E) and (**C**,**D**) periodic acid–Schiff (PAS). (**A**,**C**) PBS + FA control group: H&E staining showed extensive inflammatory cell infiltration in the dermis and subcutaneous tissue; PAS staining revealed numerous yeast-like cells (arrowheads). (**B**,**D**) SC + FA immunized group: Inflammatory infiltrates were more restricted in distribution, and fewer PAS-positive yeast-like cells were observed. (**A**,**B**) Scale bar = 250 μm; (**B**,**D**) Scale bar = 50 μm.

Since cutaneous manifestations are common in *S. globosa* infection, this study first evaluated the protective effect by subcutaneously challenging immunized mice with *S. globosa*. Skin lesions were recorded weekly for four weeks post-modeling. Measurement of lesion diameters one week post-infection showed that the median lesion diameter in the SC + FA group was 0.6 cm, compared to 1.0 cm in the PBS + FA group. Lesion diameters were smaller in the immunized group (*p* = 0.0310) (Figure 2A). During the four-week observation period, ulcers developed in 4 mice (40%) in the SC + FA group and 7 mice (70%) in the PBS + FA group. The ulcer incidence was lower in the SC + FA group, but the difference was not statistically significant (*p* = 0.3700) (Figure 2B). Notably, ulcers appeared one week later in the SC + FA group compared to the PBS + FA group. By the fourth week, one mouse in the PBS + FA group still exhibited an ulcer, whereas all ulcers in the SC + FA group have either healed or formed scabs, but the difference was not significant. Finally, fungal cells and inflammatory infiltrates were more frequently detected in the PBS + FA group (Figure 3).

### 3.3. SgtrCypB Formulated with FA Immunization Protects Against Systemic S. globosa Infection

**Figure 4:**
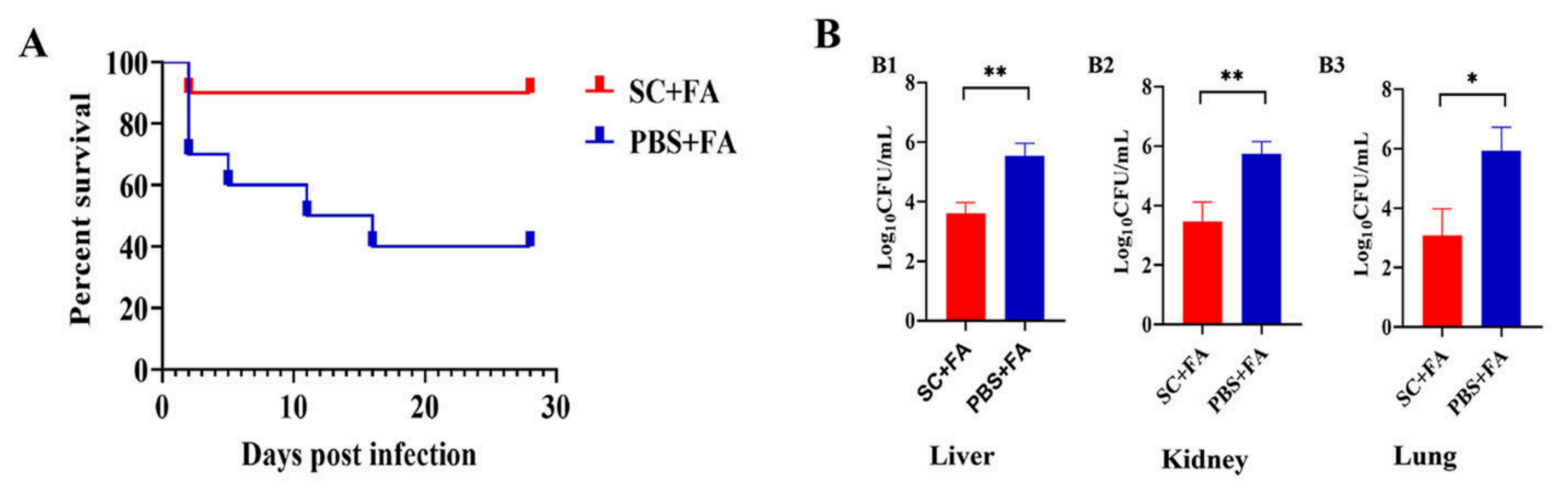
Recombinant *Sg*trCypB protein formulated with FA immunization conferred protection against systemic *S. globosa* infection in mice. Mice were immunized and challenged intravenously with 1 × 10^6^ CFU of *S. globosa*. (**A**) Survival was monitored daily for four weeks post-challenge. The SC + FA group exhibited a survival rate of 90% (9/10), whereas the PBS + FA control group had a survival rate of 40% (4/10), yielding a protection rate of 50% (*p* = 0.0246). Survival curves (*n* = 10 per group), log-rank test. (**B**) Fungal burden (log_10_ CFU/g tissue) in the (**B1**) liver, (**B2**) kidneys, and (**B3**) lungs was determined on day 5 post-infection. The SC + FA group showed significantly lower fungal loads in all three organs compared to the PBS + FA group. Data are mean ± SEM (*n* = 5 per group), Mann–Whitney U test. * *p* < 0.05, ** *p* < 0.01.

**Figure 5:**
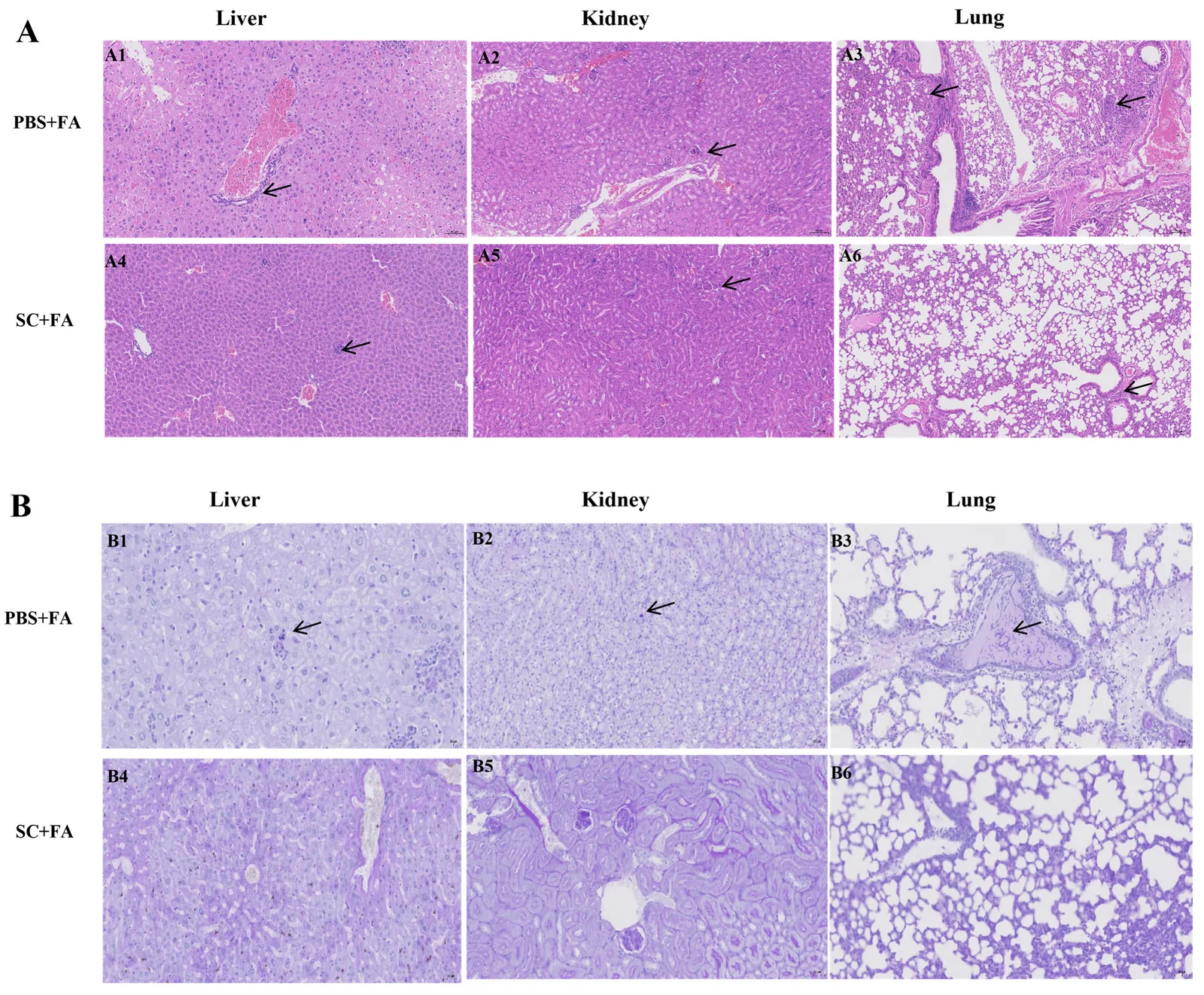
Histopathological examination of liver, kidney, and lung tissues from systemic *S. globosa* infected mice. Liver, kidney, and lung tissues were collected on day 5 post-infection and stained with (**A**) hematoxylin and eosin (H&E) and (**B**) periodic acid–Schiff (PAS). (**A1**–**A3**,**B1**–**B3**) PBS + FA control group and (**A4**–**A6**,**B4**–**B6**) SC + FA immunized group: inflammatory cell infiltration and tissue destruction were observed, with fungal cells (arrowheads) detected by PAS staining. (**A**) Scale bars = 50 μm; (**B**) Scale bars = 20 μm.

This study further explored the protective effect of recombinant *Sg*trCypB protein immunization against systemic *S. globosa* infection. The survival rate in the SC + FA group was 90%, compared to 40% in the PBS + FA group, representing a protection rate of 50% (*p* = 0.0246) (Figure 4A). Meanwhile to assess the impact of immunization on target organs, we compared the fungal burden in the liver, kidney, and lungs post-infection. At 4 weeks after infection, the fungal loads in the liver, kidney, and lung tissues of the SC + FA group were significantly lower than those in the PBS + FA group (*p* < 0.0500) (Figure 4B). Furthermore, histopathological examination of the liver, kidney, and lungs showed inflammatory responses and fungal cells, yet quantification proved difficult (Figure 5).

### 3.4. SgtrCypB Formulated with FA Immunization Confers Cross-Protection Against Systemic S. schenckii Infection

**Figure 6:**
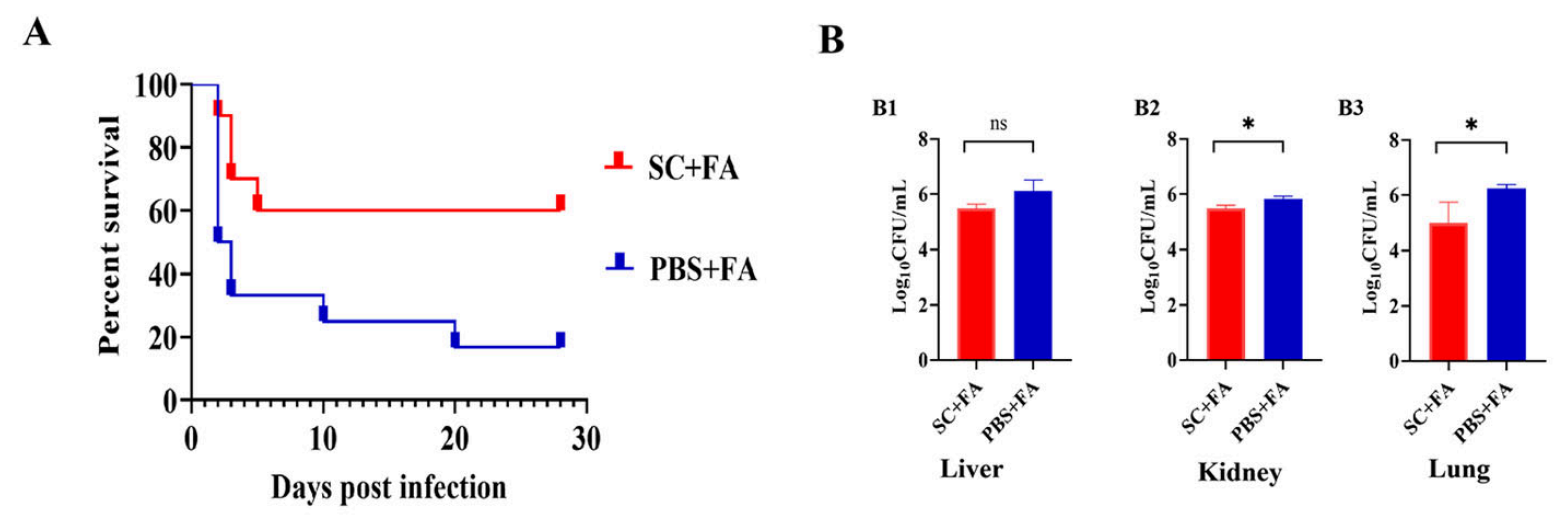
Recombinant *Sg*trCypB protein formulated with FA immunization conferred protection against systemic *S. schenckii* infection in mice. Mice were immunized and challenged intravenously with 5 × 10^5^ CFU/mL CFU of *S. schenckii*. (**A**) Survival was monitored daily for four weeks post-challenge. The SC + FA group showed a survival rate of 60% (6/10), whereas the PBS + FA control group had a survival rate of 20% (2/10), representing a protection rate of 40% (*p* = 0.033). Survival curves (*n* = 10 per group), log-rank test. (**B**) Fungal burden (log_10_CFU/g tissue) in the (**B1**) liver, (**B2**) kidneys, and (**B3**) lungs was determined on day 5 post-infection. The SC + FA group exhibited significantly lower fungal loads in the kidneys and lungs compared to the PBS + FA group (both *p* < 0.05, *n* = 5 per group). The reduction in hepatic fungal burden did not reach statistical significance (*p* = 0.0666). Data are mean ± SEM (*n* = 5 per group), Mann–Whitney U test (*, *p* < 0.05; ns, not significant).

**Figure 7:**
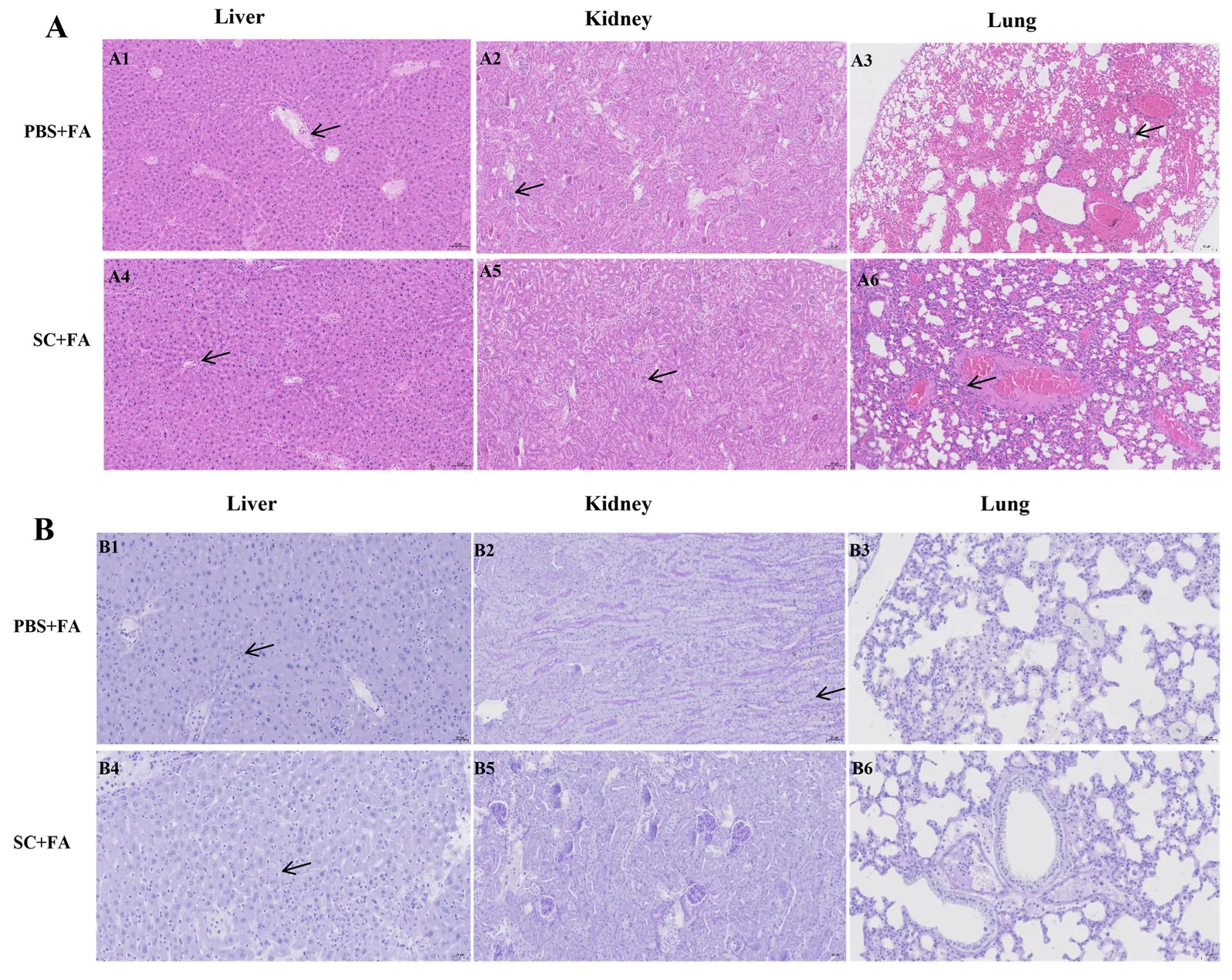
Histopathological examination of liver, kidney, and lung tissues from systemic *S. schenckii* infection mice. Liver, kidney, and lung tissues were collected on day 5 post-infection and stained with (**A**) hematoxylin and eosin (H&E) and (**B**) periodic acid–Schiff (PAS). (**A1**–**A3**,**B1**–**B3**) PBS + FA control group and (**A4**–**A6**,**B4**–**B6**) SC + FA immunized group: inflammatory cell infiltration and tissue destruction were observed, with fungal cells (arrowheads) detected by PAS staining. (**A**) Scale bars = 50 μm; (**B**) Scale bars = 20 μm.

This study also constructed a systemic *S. schenckii* infection mouse model in both the SC + FA and PBS + FA groups to assess any potential cross-protective immunity. During the four-week observation period, the survival rate for *S. schenckii* systemically infected mice was 60% in the SC + FA group and 20% in the PBS + FA group, representing a protection rate of 40% (*p* = 0.0335) (Figure 6A). The fungal burden at 4 weeks post-infection in the kidney and lungs, but not the liver, of the SC + FA group was lower than that in the PBS + FA group (*p* < 0.05; Figure 6B). Furthermore, histopathological examination of the liver, kidney, and lungs showed inflammatory responses and fungal cells, yet quantification proved difficult (Figure 7).

### 3.5. SgtrCypB Formulated with FA Immunization Provides Minimal Protection Against Systemic C. albicans Infection

**Figure 8:**
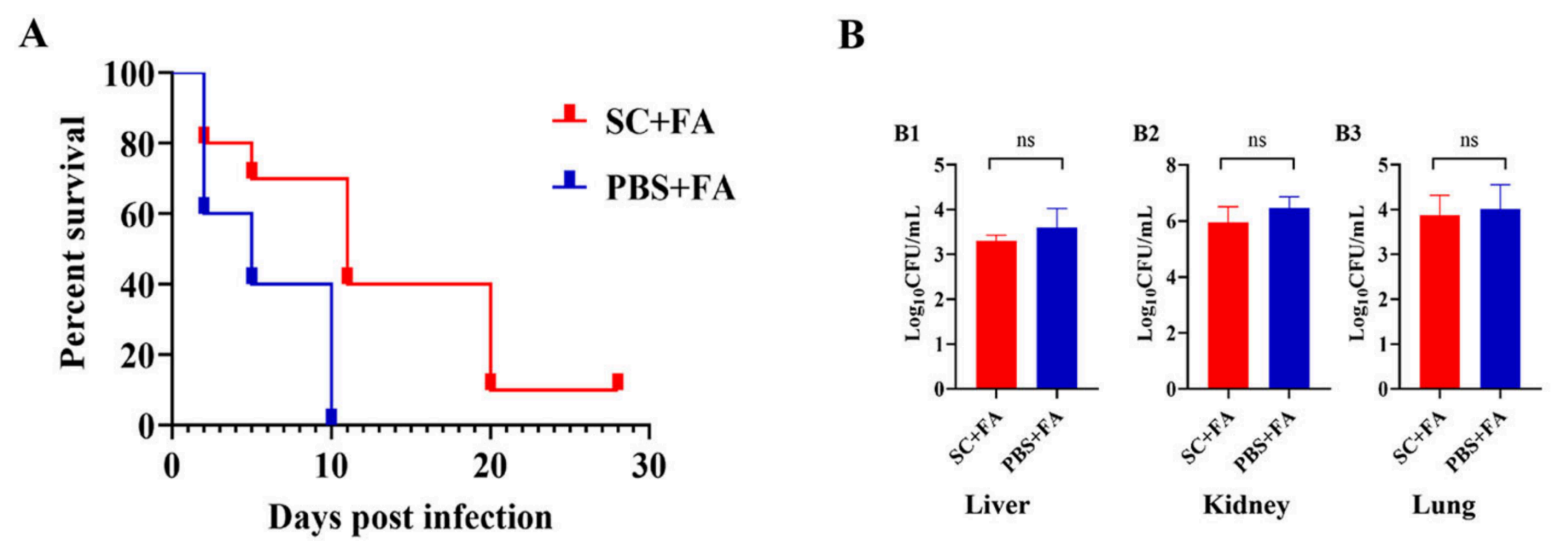
Recombinant *Sg*trCypB protein formulated with FA immunization conferred protection against systemic *C. albicans* infection in mice. Mice were immunized and challenged intravenously with 5 × 10^4^ CFU of *C. albicans*. (**A**) Survival was monitored daily for four weeks post-challenge. The SC + FA group showed a survival rate of 10% (1/10), whereas the PBS + FA control group had a survival rate of 0% (0/10), representing a protection rate of 10% (*p* = 0.0211). Survival curves (*n* = 10 per group), log-rank test. (**B**) Fungal burden (log_10_CFU/g tissue) in the (**B1**) liver, (**B2**) kidneys, and (**B3**) lungs was determined on day 5 post-infection. No statistically significant differences in fungal loads were observed between the SC + FA and PBS + FA groups for any of the three organs (all *p* > 0.05; *n* = 5 per group). Data are mean ± SEM (*n* = 5 per group), Mann–Whitney U test (ns, not significant).

**Figure 9:**
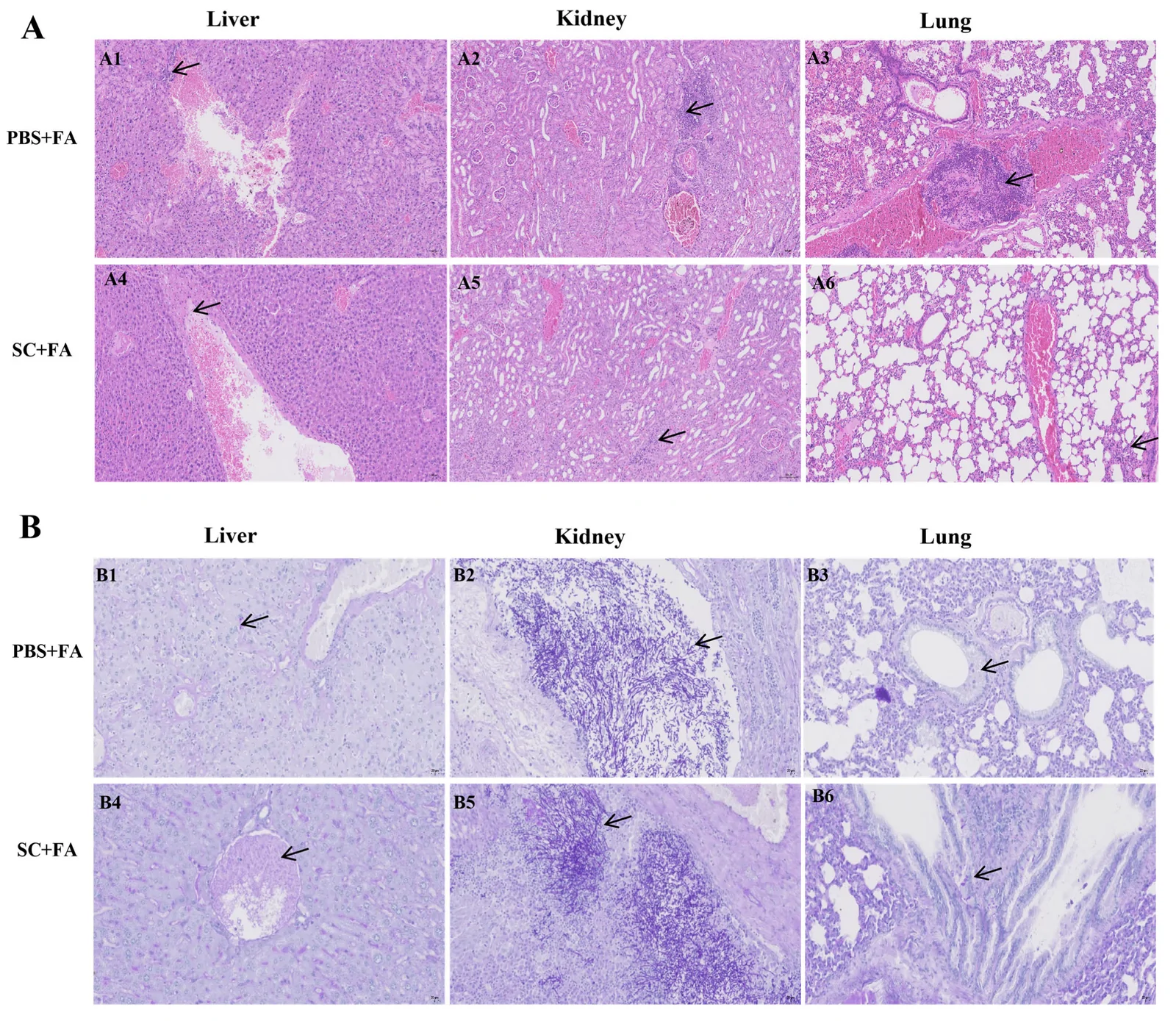
Histopathological examination of liver, kidney, and lung tissues from systemic *C. albicans* infection mice. Liver, kidney, and lung tissues were collected on day 5 post-infection and stained with (**A**) hematoxylin and eosin (H&E) and (**B**) periodic acid–Schiff (PAS). (**A1**–**A3**,**B1**–**B3**) PBS + FA control group and (**A4**–**A6**,**B4**–**B6**) SC + FA immunized group: inflammatory cell infiltration and tissue destruction were observed, with fungal cells (arrowheads) detected by PAS staining. (**A**) Scale bars = 50 μm; (**B**) Scale bars = 20 μm.

Because *C. albicans* lacks a homolog protein of *Sg*trCypB protein, we are interested to see if *Sg*trCypB protein can still immunize and protect to host against systemic *C. albicans* infection. During the four-week observation period, the survival rate for *C. albicans* systemically infected mice was 10% in the immunization group and 0% in the control group, representing a protection rate of 10% (*p* = 0.0211) (Figure 8A). Comparison of the fungal burden (CFU) in the liver, kidney, and lungs post-infection found no statistically significant differences in organ fungal loads between the groups (*p* > 0.0500) (Figure 8B). Furthermore, histopathological examination of the liver, kidney, and lungs showed inflammatory responses or fungal cells between the SC + FA and PBS + FA groups (Figure 9).

## 4. Discussion

In this study, we evaluated the immunoprotective potential of *S. globosa* cyclophilin B using a recombinant truncated protein, *Sg*trCypB. Immunization with *Sg*trCypB formulated with Freund’s adjuvant induced high-titer antibody responses and conferred protection against both cutaneous and systemic *S. globosa* infection in mice. Partial cross-protection was also observed against systemic *S. schenckii* infection, whereas protection against *C. albicans* was limited. Collectively, these findings identify cyclophilin B as a previously unrecognized protective antigen of *Sporothrix* and expand the repertoire of candidate vaccine antigens beyond the extensively studied Gp70 and enolase.

Immunization with the recombinant *Sg*trCypB protein alone elicited only a modest antibody response. Therefore, we adopted a prolonged-interval immunization protocol, which induced substantially higher antibody titers despite a lower cumulative antigen dose and a longer interval compared to the weekly regimen without adjuvant. Recombinant fungal subunit vaccines generally exhibit limited intrinsic immunogenicity to induce protective responses, particularly Th1/Th17-type cellular immunity [45,46]. Previous studies have demonstrated that compared with traditional vaccines, recombinant protein and synthetic peptide vaccines—particularly recombinant protein or synthetic peptide vaccines—offer advantages in terms of antigen definition, safety, and manufacturing consistency but generally exhibit lower immunogenicity than crude inactivated whole-cell vaccines. Their relatively low immunogenicity remains an important limitation for achieving protective immunity and underscores their critical role in vaccine development. Adjuvants can enhance humoral immunity or modulate the nature of the immune response, often allowing reductions in both antigen dose and immunization frequency while still eliciting adequate immunogenicity 47. Among the most extensively characterized and widely used adjuvants in experimental settings is Freund’s adjuvant, a stable water-in-oil emulsion prepared from paraffin oil and lanolin. Complete Freund’s adjuvant (CFA) contains heat-killed *Mycobacterium tuberculosis*, whereas incomplete Freund’s adjuvant (IFA) does not [48]. The identification of clinically acceptable adjuvants remains an important challenge and an incomplete understanding of the optimal immune response remains major barriers to fungal vaccine development. For example, recent advances in *Sporothrix* vaccine research have explored antigen selection and immunomodulation strategies. Regulatory T-cell (Treg) depletion has been reported to enhance vaccine immunogenicity and CTLA-4 targeting promoted Th1/Th17 responses [49,50]. In this study, we used a conventional adjuvant to assess the protective potential of *Sg*trCypB, with future optimization of adjuvant selection planned to improve efficacy.

*Sg*trCypB Formulation with Freund‘s adjuvant markedly enhanced antibody production, with titers reaching 1:51,200 at week 6. This titer is notable, as serum IgG titers in sporotrichosis patients have been reported to reach up to 1:65,200 [51], suggesting that the 1:51,200 titer achieved in immunized mice fell within a range that might be immunologically meaningful in the context of human disease. Passive transfer of antigen-specific antibodies has been directly demonstrated to confer protection against *Sporothrix* infection in murine models, supporting the notion that antibody-mediated immunity might contribute to the observed protective effects. The progressive increase in titers after each boost further indicates effective priming and the generation of immunological memory [52,53]. While antibody titers correlated with reduced fungal burden in our study, formal demonstration of causality—through passive transfer or antibody depletion experiments—remains to be established. However, the immunization schedules and cumulative antigen doses differed between the antigen-alone pilot study and the adjuvant-formulated main experiment, precluding direct comparison. The antigen-alone data should be interpreted as preliminary evidence of the protein’s limited intrinsic immunogenicity, not as a comparator to the adjuvanted formulation. The PBS + FA group served as the primary control to account for the non-specific immunostimulatory effects of Freund‘s adjuvant. Given that the core question was whether *Sg*trCypB confers protection beyond adjuvant-induced effects, the PBS + FA group represented the most appropriate comparator in this proof-of-concept study. A PBS-only group would have provided additional baseline information for ELISA cut-off determination, and this should be considered in future studies.

*Sg*trCypB formulated with FA demonstrated measurable protection in both cutaneous (reduced lesion size) and systemic (90% survival) models of *S. globosa* infection. Quantifying vaccine efficacy in cutaneous sporotrichosis models has been challenging, and previous studies are scarce. One earlier study evaluated Gp60 immunization against cutaneous *S. schenckii* infection and reported limited protective effects, with no significant difference in ulcer incidence in C57BL/6 mice and comparable inflammatory responses in BALB/c mice [23]. Notably, that study did not employ PAS staining to assess fungal burden in tissues. In contrast, our study incorporated PAS staining to allowed visualization of fungal elements. It should be noted, however, that the difference in ulcer incidence between the SC + FA and PBS + FA groups (40% vs. 70%) did not reach statistical significance (*p* = 0.3700). First, the relatively small sample size may have limited the statistical power to detect a true difference in this binary outcome. Second, ulcer formation represents a relatively severe and late-stage manifestation of cutaneous infection, which may be more variable and less sensitive to vaccine-induced protection than earlier measures such as lesion diameter.

In the systemic *S. globosa* infection model, *Sg*trCypB formulated with FA immunization conferred 90% survival (vs. 40% in controls) and significantly reduced fungal burden in the liver, kidney, and lung. The direct survival percentages, while lower than the 60% reported for a phage-displayed Gp70 epitope vaccine [27], might reflect differences in antigen format and challenge models. Importantly, Unlike a single displayed epitope, recombinant SgtrCypB contains multiple potential B- and T-cell epitopes within the retained functional region, which may contribute to broader antigen recognition—a hypothesis warranting future investigation. For the phage-displayed Gp70 epitope vaccine, bacteriophages exhibit intrinsic adjuvant properties capable of stimulating innate immune responses through the activation of Toll-like receptors (TLRs) and other pattern-recognition receptors (PRRs) [54]. CpG-containing phage DNA may activate TLR9 signaling and their highly ordered capsid architecture can promote efficient B-cell receptor cross-linking [55]. Following uptake by antigen-presenting cells, phage-associated antigens can be processed and presented through both MHC class I and II pathways, enabling concurrent activation of CD8^+^ cytotoxic T lymphocytes and CD4^+^ helper T cells, thereby eliciting a broad repertoire of Th1, Th2, and Th17 responses [56]. The Gp70 phage vaccine’s 60% protection rate reflects not only the epitope itself but also the self-adjuvating properties of the phage carrier. The moderate protection rate in our study (50%) therefore should not be attributed solely to intrinsic antigenicity of cyclophilin B, but rather as a consequence of different delivery systems and adjuvant dependence. This comparison underscores that the choice of delivery platform—not just the antigen—critically determines vaccine efficacy, and highlights the need for optimizing *Sg*trCypB formulation with clinically applicable adjuvants to fully realize its protective potential.

*Sg*trCypB exhibited species-specific protective immunity against *S. globosa* and partial cross-protection against *Sporothrix schenckii*. The partial cross-protection observed against *S. schenckii* (60% survival vs. 20% in controls) was suggesting that conserved sequence homology (approximately 99%) between *S. globosa* and *S. schenckii* cyclophilin B [33], resulting in cross-reactivity of protective B- and possibly T-cell epitopes between the two *Sporothrix* species. In contrast, *C. albicans* lacks a closely related homolog of *Sg*CypB, so the cautious 10% protection could originate from low-affinity cross-reactive antibodies or from nonspecific immunostimulatory properties of the recombinant protein or Freund’s adjuvant. A cyclophilin B-based antigen might confer broad protection against *S. globosa* and *S. schenckii*. Nevertheless, the precise mechanism for the differential cross-protection requires further epitope-mapping and cross-recognition studies. The substantial disease burden and poor prognosis of sporotrichosis in immunocompromised populations underscore the translational potential of our findings [11,57,58]. The significant improvement in survival following immunization supports further development of this vaccine strategy.

The protective immunity conferred by *Sg*trCypB formulated with FA may involve both humoral and cellular immune mechanisms. Cyclophilins from various organisms have demonstrated immunomodulatory properties: *Schistosoma japonicum* cyclophilin A promotes Th2 responses [59,60], and human cyclophilin B induces integrin-mediated adhesion of CD4^+^ T lymphocytes and monocytes/macrophages to fibronectin, while also activating memory CD4^+^ T cells and macrophages [61]. Our previous work showed *S. globosa* cyclophilin B is localized to the extracellular side of the fungus [33], potentially facilitating recognition. When delivered with Freund’s adjuvant (CFA/IFA), *Sg*trCypB likely elicits both humoral and cellular immunity. The sequential CFA/IFA protocol employed in this study—CFA for primary immunization followed by IFA boosts—is known to establish a Th1-biased response through the mycobacterial components in CFA, which is subsequently maintained by IFA boosts. However, IFA itself also contributes to a balanced Th1/Th2 response and antibody production. Thus, while the overall response is Th1-skewed, Th2-associated mechanisms may also contribute to the observed high-titer antibody responses [62,63]. This is consistent with studies showing that Freund‘s adjuvant can induce strong IgG1 antibody responses [64,65], and with reports that protective immunity against sporotrichosis can involve a mixed Th1/Th2/Th17 cytokine profile [66,67]. Direct cytokine profiling and T-cell subset analysis will be required to confirm the exact correlates of protection.

Several limitations should be acknowledged, and future studies are warranted to address these gaps and to advance *Sg*trCypB toward clinical translation. First, the use of Freund’s adjuvant, while effective for proof-of-concept, is not clinically translatable due to its toxicity (ulcers, granulomas) [27,30]. Future studies should evaluate clinically acceptable adjuvant systems capable of inducing balanced Th1/Th17 responses, which are considered important for antifungal immunity. Second, although an antigen-only regimen was evaluated for immunogenicity, the infection-challenge experiments primarily compared the *Sg*trCypB + FA and PBS + FA groups, which limits our ability to definitively attribute the observed protection solely to the recombinant protein. However, our preliminary data showed that immunization with *Sg*trCypB without adjuvant elicited only minimal antibody responses, supporting the necessity of adjuvant for immunogenicity. Third, we acknowledge that histopathological evaluation was primarily descriptive. Nevertheless, quantitative CFU measurements provided a more direct and comparable measure of fungal burden across organs, which consistently supported the protective effects observed in immunized mice, and uninfected groups (PBS-only or PBS + FA-without-infection) were not included to establish the reference histological appearance of normal tissue or to assess the inflammatory effects of the adjuvant in isolation. The future studies should include appropriate uninfected controls for a more comprehensive histopathological evaluation. Fourth, cross-protection against *S. schenckii* was evaluated using only one strain; further testing against additional strains and species, particularly *S. brasiliensis*, would strengthen the evidence for broad-spectrum protection. While we observed high antibody titers were associated with protection, the study did not directly assess cellular immune responses or the protective role of specific antibody subclasses. Future mechanistic studies, including T-cell profiling and passive transfer experiments, will be necessary to establish the correlates of protection. Finally, safety evaluation, including body weight monitoring and autoantibody screening, was beyond the scope of this proof-of-concept study and should be systematically assessed in future work, as emphasized in recent reviews on experimental sporotrichosis models [68]. Direct comparison with antifungal therapy was beyond the scope of this study. However, a vaccine strategy could complement existing treatments by providing prevention, reducing the need for long-term therapy, and addressing drug resistance and recurrence in high-risk populations.

## 5. Conclusions

This study identified *Sg*trCypB as a promising protective antigen against *Sporothrix* infection, conferred protection in both cutaneous and systemic murine models. The protective effects, however, were dependent on the complete formulation with Freund’s adjuvant, as immunization with the recombinant protein alone failed to induce sufficient antibody responses. Partial cross-protection against *S. schenckii* was observed, consistent with the high sequence similarity between cyclophilins of the two species, while minimal protection were detected against *C. albicans*, supporting the specificity of the immune response. Collectively, these findings have identified cyclophilin B as a promising antigen for further vaccine development. Given the endemicity of sporotrichosis in China and Latin America and the limitations of current antifungal therapy, continued optimization of adjuvant selection and mechanistic understanding will be essential to translate this candidate into clinical application.

## Data Availability

The original contributions presented in this study are included in the article. Further inquiries can be directed to the corresponding authors.

## Supplementary Materials

The following supporting information can be downloaded at: https://www.mdpi.com/article/10.3390/jof12090706/s1, Figure S1: Sequence alignment of *Sg*trCypB and *Mycobacterium tuberculosis* PpiB.Amino acidsequences of *Sg*trCypB (*Sporothrix globosa* cyclophilin B) and PpiB (Rv2582) from *Mycobacterium tuberculosis* H37Rv were aligned using Basic Local Alignment Search Tool for proteins (BLASTp; National Center for Biotechnology Information, Bethesda, MD, USA). The overall sequence identity between the two proteins was below 35%.
