# Supplementary material for: SgtrCypB Formulated with Freund’s Adjuvant Confers Protective Immunity Against S. globosa and Partial Cross-Protection Against S. schenckii Infections in Mice
Source: J Fungi (Basel). 2026 Sep 21;12(9):706. doi: 10.3390/jof12090706 (PMC13608398; doi:10.3390/jof12090706)
Supplement: Supplementary file 1 [file jof-12-00706-s001.zip › jof-4502918-supplementary.pdf]

Alignment statistics for match #1

Score   Expect Method   Identities   Positives Gaps

65.9 bits(159)   3e-17 Compositional matrix adjust.   55/160(34%)   81/160(50%) 21/160(13%)

```
Query  24   GRIVMGLYGKTVPETVENFRALATGEKGFGYSGSTFHRVIKDFMI---QGGDFTRGDGTG  80
            G  I+L     P TV +F +LA  ++GF + G+T HR+    M+   Q GD  +GDGTG
Sbjct  134  GNIGLMLANNESPCTVNSFVSLA--QQGF-FKGTTCHRLTTSPMLAVLQCGD-PKGDGTG  189

Query  81   GKSI-YGDRFKDENFKLKHTK--------KGMLSMANAGKDTNGSQFFITTAITSWLDGR  131
            G    + + +  + +     K        +G L+MANAG +TN SQFF+     S L  +
Sbjct  190  GPGYQFANEYPTDQYSANDPKLNEPVIYPRGTLAMANAGPNTNSSQFFMVYR-DSKLPPQ  248

Query  132  HVVFGEV-LEGYDIVDKIQN--VKKGAQD-RPTEAVKITE  167
            + VFG+  +G   +DKI    V  G+D +P   V IT
Sbjct  249  YTVFGTIQADGLTTLDKIAKAGVAGGGEDGKPATEVTITS  288
```

Supplementary Figure S1. Sequence alignment of *Sg*trCypB and *Mycobacterium tuberculosis* PpiB.Amino acid sequences of *Sg*trCypB (*Sporothrix globosa* cyclophilin B) and PpiB (Rv2582) from *Mycobacterium tuberculosis* H37Rv were aligned using Basic Local Alignment Search Tool for proteins (BLASTp; National Center for Biotechnology Information, Bethesda, MD, USA). The overall sequence identity between the two proteins was below 35%.
